# Evaluating the Role of Urinary Amylase in Diagnosing Acute Pancreatitis: A Systematic Review and Meta-Analysis

**DOI:** 10.7759/cureus.98982

**Published:** 2025-12-11

**Authors:** Cathleen A McCarrick, Ashokkumar Singaravelu, Ahmed Al Rasheed, Ronan Murphy

**Affiliations:** 1 Surgery, University College Dublin, Dublin, IRL; 2 Emergency Medicine, Royal College of Surgeons in Ireland, Dublin, IRL; 3 Emergency Medicine, University Hospital Kerry, Tralee, IRL

**Keywords:** acute pancreatitis, diagnostic accuracy urinary amylase, diagnostic threshold urinary amylase, pancreatitis, sensitivity, specificity, urinary amylase

## Abstract

Acute pancreatitis is a life-threatening and increasingly common condition. Although serum amylase and lipase are established diagnostic markers, the clinical relevance of urinary amylase is still uncertain. This systematic review and meta-analysis aimed to assess the diagnostic accuracy of urinary amylase in adults with suspected acute pancreatitis. We registered our review with PROSPERO (CRD42024528325) and adhered to the Preferred Reporting Items for Systematic Reviews and Meta-Analyses (PRISMA) reporting guidelines. We systematically searched PubMed, EMBASE, Ovid Medline, Scopus, the Cochrane Library, and Web of Science for studies of adult patients with acute abdominal pain who had quantitative urinary amylase measurements performed. Two reviewers independently screened articles for inclusion and extracted data from eligible studies. The risk of bias and applicability of the included studies were assessed using the Quality Assessment of Diagnostic Accuracy Studies (QUADAS-2) tool. Our search identified 178 studies, of which eight met the inclusion criteria; three cross-sectional studies and two case-control studies were included in the meta-analysis. Urinary amylase had a sensitivity of 0.80 (95% confidence interval (CI): 0.65-0.89) and specificity of 0.86 (95% CI: 0.76-0.93) at a model-derived optimal threshold of 1,373 IU/L. Our results indicate that urinary amylase does not appear to outperform serum amylase, and its suggested utility in delayed or atypical presentations of acute pancreatitis remains unsubstantiated.

## Introduction and background

Pancreatitis is a severe disease associated with substantial morbidity and mortality, and its incidence continues to rise globally [1]. According to the revised Atlanta Classification (2012) [2], the diagnosis of acute pancreatitis requires at least two of the following criteria: (1) characteristic upper abdominal pain; (2) serum amylase or lipase levels exceeding three times the upper limit of normal; and (3) typical imaging features. However, clinicians occasionally encounter patients with suspected acute pancreatitis who present with normal or borderline enzyme levels [3,4]. Because urinary amylase may remain elevated longer after symptom onset, it has been proposed as a potential diagnostic tool in such cases, although its accuracy and clinical value remain uncertain.

Although elevated serum amylase or lipase levels (greater than three times the upper limit of normal) can support the diagnosis of acute pancreatitis, they are not pathognomonic [5]. These enzymes are not useful for assessing the severity of acute pancreatitis nor monitoring its progress or resolution [6]. Additionally, although the amylase-creatinine clearance ratio has been investigated, it has not proven useful in routine clinical practice for diagnosing acute pancreatitis [7,8]. Physiological phenomena, multiple diverse intra- and extra-abdominal pathologies, and pharmaceutical agents can all raise pancreatic enzyme levels, potentially leading to false positives when evaluating for acute pancreatitis [9-12]. Conversely, conditions such as exocrine pancreatic insufficiency (e.g., chronic alcohol abuse) and hypertriglyceridemia, along with the timing of enzyme sampling (very early or late presentations), can reduce the sensitivity of these markers for acute pancreatitis [13-16].

In an episode of acute pancreatitis, serum amylase levels rise in three to six hours and can normalise within 24 hours [17,18]. Elevated urinary amylase usually follows elevated serum amylase levels due to altered renal tubular reabsorption and remains elevated for 7-10 days longer than serum amylase, which is the first pancreatic enzyme to normalise [19-21]. Macro-amylasemia (characterised by a complex of immunoglobulin and normal amylase) occurs in approximately 2.5% of hyperamylasemic patients and is associated with conditions such as celiac disease, lymphoma, HIV, monoclonal gammopathy, rheumatoid arthritis, and ulcerative colitis [22]. Elevated urinary amylase effectively rules out macroamylasemia as the cause of a raised serum amylase [23].

This systematic review and meta-analysis primarily aims to assess the accuracy (sensitivity, specificity, optimal threshold) of urinary amylase in the diagnosis of acute pancreatitis in adults presenting with acute abdominal pain. The secondary objective is to evaluate its potential role within the overall diagnostic pathway.

## Review

Methodology

This systematic review and meta-analysis were registered with the International Prospective Register of Systematic Reviews (PROSPERO, registration number: CRD42024528325) and reported in accordance with the Preferred Reporting Items for Systematic Reviews and Meta-Analyses (PRISMA) guidelines [24].

Data Sources

We performed a systematic search of PubMed, EMBASE, Ovid Medline, Scopus, Cochrane Library, and Web of Science databases without applying any date restrictions. We also searched the clinical trials registry ClinicalTrials.gov and the health sciences preprint server medRxiv.org, though no studies combining urinary amylase and acute pancreatitis were identified in either source. The final search of all databases was carried out on 28 September 2024. In addition, reference lists of included studies were screened to identify any relevant studies that may have been missed in the database searches.

Search Strategy

Table 1 presents the details of our search strategy. No filters were applied to any of the database searches. The inclusion criteria were adult patients presenting with acute abdominal pain who underwent quantitative spot urinary amylase testing, reported in IU/L. The exclusion criteria were as follows: paediatric patients, pregnant patients, study language other than English, and studies not conforming to case-control, cross-sectional, or diagnostic randomised controlled trial designs.

**Table 1:** Database search strategy

Database	Search strategy
PubMed	(("Acute Pancreatitis" OR "Pancreatitis"[Mesh:NoExp]) AND ("urinary amylase")) AND (sensitivity OR specificity)
Ovid Medline	((sensitivity or specificity) and urinary amylase).mp. and (acute pancreatitis.mp. or exp pancreatitis/)
Scopus	(TITLE-ABS-KEY (urinary AND amylase) AND TITLE-ABS-KEY (acute AND pancreatitis) AND TITLE-ABS-KEY (sensitivity OR specificity))
Embase	(urinary AND ('amylase'/exp OR amylase)) AND (sensitivity OR specificity) AND ('acute pancreatitis'/exp OR 'acute pancreatitis')
Cochrane Database	"urinary amylase" AND "acute pancreatitis" AND (sensitivity OR specificity) TITLE-ABSTRACT-KEYWORD

**Figure 1:**
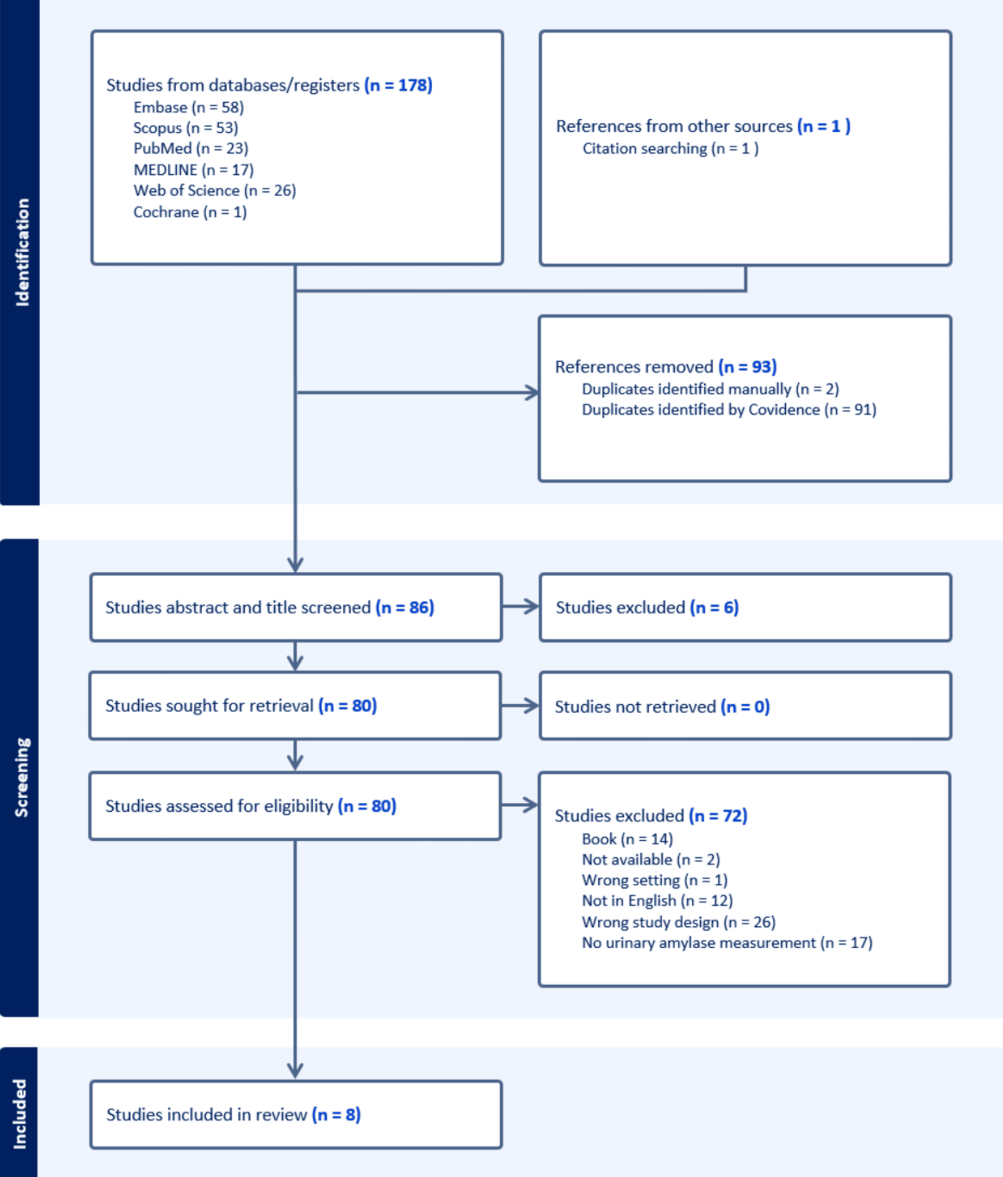
PRISMA flow diagram depicting the database search results PRISMA: Preferred Reporting Items for Systematic Reviews and Meta-Analyses

Study selection: Using Covidence systematic review software (Figure 1), two researchers (RM and CM) independently screened titles and abstracts for relevance during the initial phase. Articles deemed potentially eligible were then assessed through full-text review. A third independent reviewer was available to resolve disagreements, although this was not necessary. We considered diagnostic randomised trials, cross-sectional studies, and case-control studies. An additional practicing Emergency Medicine Physician (AA) assisted in the derivation of the search strategy for each database and review of the write-up for general readability and content relevance to acute care providers.

Data extraction: From each included study, two researchers (RM and CM) independently extracted data to evaluate study quality, assess applicability, and perform necessary statistical analyses in collaboration with authors AS and AA. The prespecified variables for extraction included: authors, year of publication, geographic location, sample size, study design, inclusion and exclusion criteria, timing of urine sample collection, diagnostic thresholds for urinary amylase (IU/L), reference standard for diagnosing acute pancreatitis, and the numbers of true positives, false positives, false negatives, and true negatives. When additional information was needed during full-text review, study authors were contacted directly.

Appraisal of methodological quality: Studies were assessed for methodological quality using the Quality Assessment of Diagnostic Accuracy Studies (QUADAS-2) tool for diagnostic accuracy studies [25]. Two researchers (RM and CM) conducted these assessments, with a third reviewer available if consensus was needed, though their input was not required.

Risk of bias was evaluated across four domains: patient selection, index test, reference standard, and flow and timing.

Patient selection, in terms of random or consecutive sample enrollment, the avoidance of a case-control study design, and the avoidance of inappropriate exclusions.

Index test, quantitative (spot) urinary amylase (IU/L) in terms of its being interpreted without knowledge of the reference standard, and a threshold being prespecified when used. Of note, amylase often forms part of the reference standard for diagnosing acute pancreatitis, potentially creating incorporation bias that will tend to overestimate the diagnostic performance of urinary amylase.

Reference standard, in terms of its likelihood of correctly identifying acute pancreatitis by presenting sufficient detail on what the standard comprised (e.g, clinical assessment, enzyme type(s), and threshold specified, radiological or surgical components) and its interpretation being separate from the index test. 

Flow and timing, in terms of the index urine test and the reference serum test(s) being conducted at the same time (samples being taken from the patient on presentation and before diagnosis for this review), all patients received the same reference standard, and all patients were included in the analysis.

Applicability was assessed over three domains: patient selection, index test, and reference standard.

Patient selection, in terms of the selected patients being acute hospital presentations, and including patients who were symptomatic with diseases other than acute pancreatitis, to ensure a discriminating assessment of the index test.

Index test, quantitative (spot) urinary amylase, is being conducted on the patient presentation to the hospital.

Reference standard, in terms of its alignment with an appropriate reference standard for the diagnosis of acute pancreatitis (e.g, two of three: clinical findings suspicious based on history and physical examination, raised enzyme tests above pre-specified diagnostic threshold, imaging findings).

Main outcomes and measures: The primary outcome was the sensitivity and specificity of urinary amylase for the diagnosis of acute pancreatitis.

Statistical Analysis

We included compatible diagnostic cross-sectional and case-control studies that reported sufficient data to construct 2x2 tables for urinary amylase versus the diagnostic reference standard for acute pancreatitis in our meta-analysis. Analysis of publication bias and heterogeneity was performed using the RStudio 2024.12.1 package *meta* [26]. Thereafter, a meta-analysis was performed using a random effects model and the data presented in forest plots [27]. Spearman correlation Analysis between sensitivity and false positive rate was performed to determine the presence of a threshold effect.

Finally, a bivariate linear mixed effects model described by Steinhauser [28] was employed, generating a summary receiver operating characteristic (SROC) curve with pooled sensitivity and specificity calculated at each specified threshold in the included studies using the RStudio 2024.12.1 package *diagmeta*. This model is appropriate here as it best allows for multiple thresholds per study and accounts for any threshold effects. An optimal threshold across studies was found using maximisation of the Youden Index. The Youden index is a well-established method to indicate an optimal cut-off point for distinguishing between two groups, balancing sensitivity and specificity [29]. 

Results

Search Results

Characteristics of the included studies: Of the 178 records identified, 93 duplicates were removed, 86 records screened, and eight studies met criteria for inclusion in the systematic review (of which five were included in meta-analysis). Table 2 presents the main characteristics of all included studies. Table 3 details the data extracted from the studies included in the meta-analysis. Table 4 outlines their quality assessments.

**Table 2:** Study characteristics CT: computed tomography; US: ultrasound

Study	Setting	Country	N	Inclusion criteria	Exclusion criteria	Diagnostic criteria	Symptom duration	Design	Urinary amylase diagnostic thresholds
Hedström et al., 1996 [30]	University teaching hospital	Finland	101	Acute abdominal pain	None	Clinical and serum amylase >300 IU/L and CT findings	Unspecified	Case-control	3200 IU/L, 3700IU/L
Hedström et al., 1998 [31]	University teaching hospital	Finland	500	Acute abdominal pain	None	Clinical and serum amylase >300 IU/L or urinary amylase >2000 IU/L and/or CT or US findings	Unspecified	Cross-sectional	2000 IU/L, 3960 IU/L, 6000 IU/L
Pezzilli et al., 2001 [32]	University teaching hospital	Italy	90	Acute abdominal pain	None	Clinical and CT or US findings	0-3 days	Case-control	700 IU/L
Treacy et al., 2001 [33]	University teaching hospital	Australia	86	Acute abdominal pain	Patients with an alternative diagnosis or an indeterminate diagnosis	Clinical, imaging findings, operative or endoscopic findings, and autopsy findings	Unspecified	Cross-sectional	550 IU/L
Kemppainen et al., 1997 [34]	University teaching hospital	Finland	500	Acute abdominal pain	None	Clinical and serum amylase >300 IU/L or urinary amylase >2000 IU/L and CT or US findings	0-4 days	Cross-sectional	2000 IU/L 3140 IU/L 6000 IU/L
Houry et al., 1989 [35]	Acute hospital	France	100	Acute abdominal pain	None	Clinical amylase creatinine clearance ratio greater than or equal to 0.05	0-2 days	Cross-sectional	Above upper limit of normal (unspecified)
Husayni et al., 2022 [36]	University teaching hospital	India	160	Acute pancreatitis, cases and controls	Patients who did not consent to participate	Unclear	Unspecified	Case-control	306 IU/L
Kalaiventhan et al., 2024 [37]	University teaching hospital	India	220	Acute pancreatitis, cases and controls	Non-consenting patients, those with poorly controlled blood sugar, diabetes, hypertension, and chronic renal disease	Clinical, radiological, and investigative means. Unspecified	Unspecified	Case-control	None specified

**Table 3:** Meta-analysis study data TP: true positive; FP: false positive; FN: false negative; TN: true negative; CI: confidence interval

Study	Threshold (I/UL)	TP	FP	FN	TN	Total, n	Lower CI	Sensitivity	Upper CI	Youden	Lower CI	Specificity	Upper CI
Hedström et al. (1996) [30]	3200	40	4	19	38	101	0.54	0.68	0.79	0.582728	0.77	0.9	0.97
Hedström et al., J (1996) [30]	3700	35	2	24	40	101	0.46	0.59	0.72	0.545601	0.84	0.95	0.99
Kemppainen et al. (1997) [34]	2000	44	54	9	393	500	0.7	0.83	0.92	0.709383	0.85	0.88	0.91
Kemppainen et al. (1997) [34]	3140	39	22	14	425	500	0.6	0.74	0.85	0.686632	0.93	0.95	0.97
Kemppainen et al. (1997) [34]	6000	27	13	26	434	500	0.37	0.51	0.65	0.480351	0.95	0.97	0.98
Hedström et al. (1998) [31]	2000	42	54	10	394	500	0.67	0.81	0.9	0.687157	0.85	0.88	0.91
Hedström et al. (1998) [31]	3960	28	13	24	435	500	0.39	0.54	0.68	0.509444	0.95	0.97	0.98
Hedström et al. (1998) [31]	6000	22	9	30	439	500	0.29	0.42	0.57	0.402988	0.96	0.98	0.99
Pezzili et al. (2001) [32]	700	21	8	9	52	90	0.51	0.7	0.85	0.5667	0.83	0.97	1
Treacy et al. (2001) [33]	550	32	1	19	34	86	0.48	0.63	0.76	0.59888	0.85	0.97	1

**Table 4:** Quality assessment of all studies HR: high risk; LR: low risk

Study	Risk of bias				Applicability concerns		
	Patient selection	Index test	Reference standard	Flow and timing	Patient selection	Index test	Reference standard
Houry et al., 1989 [35]	LR	HR	HR	LR	LR	LR	HR
Hedström et al., 1996 [30]	HR	HR	LR	LR	LR	LR	LR
Hedström et al., 1998 [31]	LR	HR	HR	LR	LR	LR	LR
Pezzelli et al., 2001 [32]	HR	LR	LR	LR	LR	LR	LR
Treacy et al., 2001 [33]	LR	LR	LR	LR	LR	LR	LR
Kemppainen et al., 1997 [34]	LR	HR	HR	LR	LR	LR	LR
Husayni et al., 2022 [36]	HR	HR	LR	HR	HR	LR	LR
Kalaiventhan et al., 2024 [37]	HR	HR	LR	HR	HR	LR	LR

Analysis of publication bias and heterogeneity: The diagnostic odds ratio (DOR) is calculated as:

$\text{DOR} = \frac{\text{TP} / \text{FN}}{\text{FP} / \text{TN}} = \frac{\text{TP} \times \text{TN}}{\text{FP} \times \text{FN}}$

Deeks' funnel plot test for DOR indicated no significant publication bias.

Test result: t = -0.73, df = 8, p-value = 0.4873

There was no significant heterogeneity. It is notable, however, that zero heterogeneity in diagnostic test accuracy studies is unusual and likely reflects a small number of studies and amylase forming part of the reference standard. 

Quantifying heterogeneity: tau^2 = 0 [0.0000; 0.0342]; tau = 0 [0.0000; 0.1850]; I^2 = 0.0% [0.0%; 62.4%]; H = 1.00 [1.00; 1.63]

Test of heterogeneity: Q = 3.13; d.f. = 9; p-value = 0.9590

**Figure 2:**
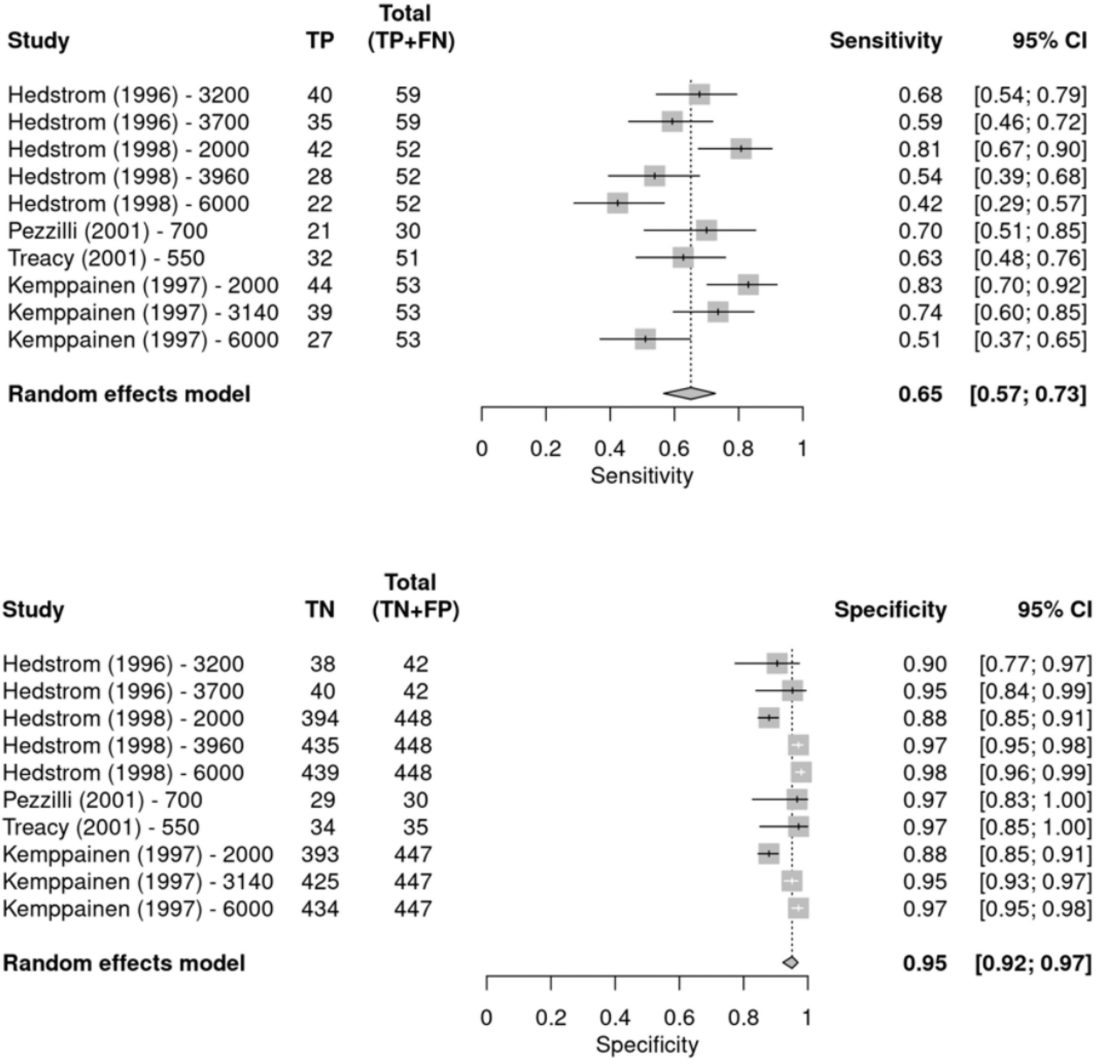
Forest plots of sensitivity and specificity for urinary amylase at various thresholds across included studies TP: true positive; FP: false positive; FN: false negative; TN: true negative; CI: confidence interval Hedstrom et al., 1996 [30]; Hedstrom et al., 1998 [31]; Pezzilli et al., 2001 [32]; Treacy et al., 2001 [33]; Kemppainen et al., 1997 [34]

Meta-analysis of diagnostic test accuracy: Three cross-sectional and two case-control studies were included in the meta-analysis [30-34]. Figure 2 shows the forest plots of sensitivity and specificity for each study at the various thresholds (IU/L). The two case-control studies scored high risk of bias in the domain of patient selection. They did use symptomatic patients as controls and characterised the study participants in good detail. In two studies, the reference standard scored a high risk of bias due to its interpretation not being separate from the index test.

Spearman correlation analysis between sensitivity and false positive rate demonstrates a significant threshold effect (r = 0.823, p = 0.01). This suggests that the sensitivity and specificity of the test vary with variation in the threshold. In the meta-analysis, the bivariate linear mixed effects model described by Steinhauser et al. [28] was also employed. This model allows for multiple threshold values per study to be included and accounts for the dependence of sensitivity and specificity on each other and threshold effects, features not addressed by the univariate meta-analysis of sensitivity and specificity shown in Figure 2.

**Figure 3:**
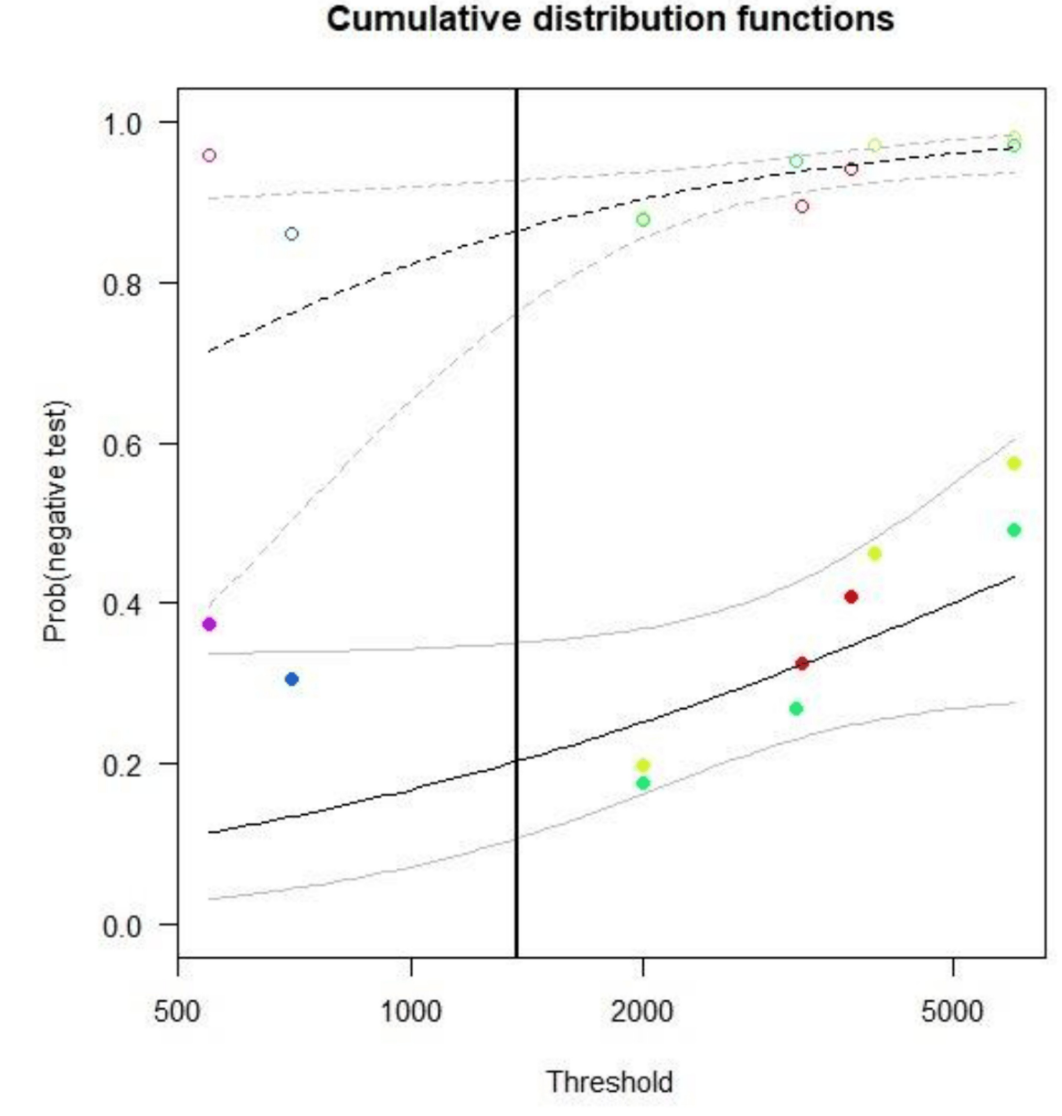
Estimated distribution functions for the non-pancreatitis individuals (open circles, dashed line) and the acute pancreatitis individuals (filled circles, solid line)

Figure 3 displays the estimated distribution functions for the non-pancreatitis individuals (open circles, dashed line) and the acute pancreatitis individuals (filled circles, solid line). The grey lines (dashed and solid, respectively) mark the confidence regions. The optimum threshold, derived from optimisation of the Youden Index, is shown as a vertical black solid line.

**Figure 4:**
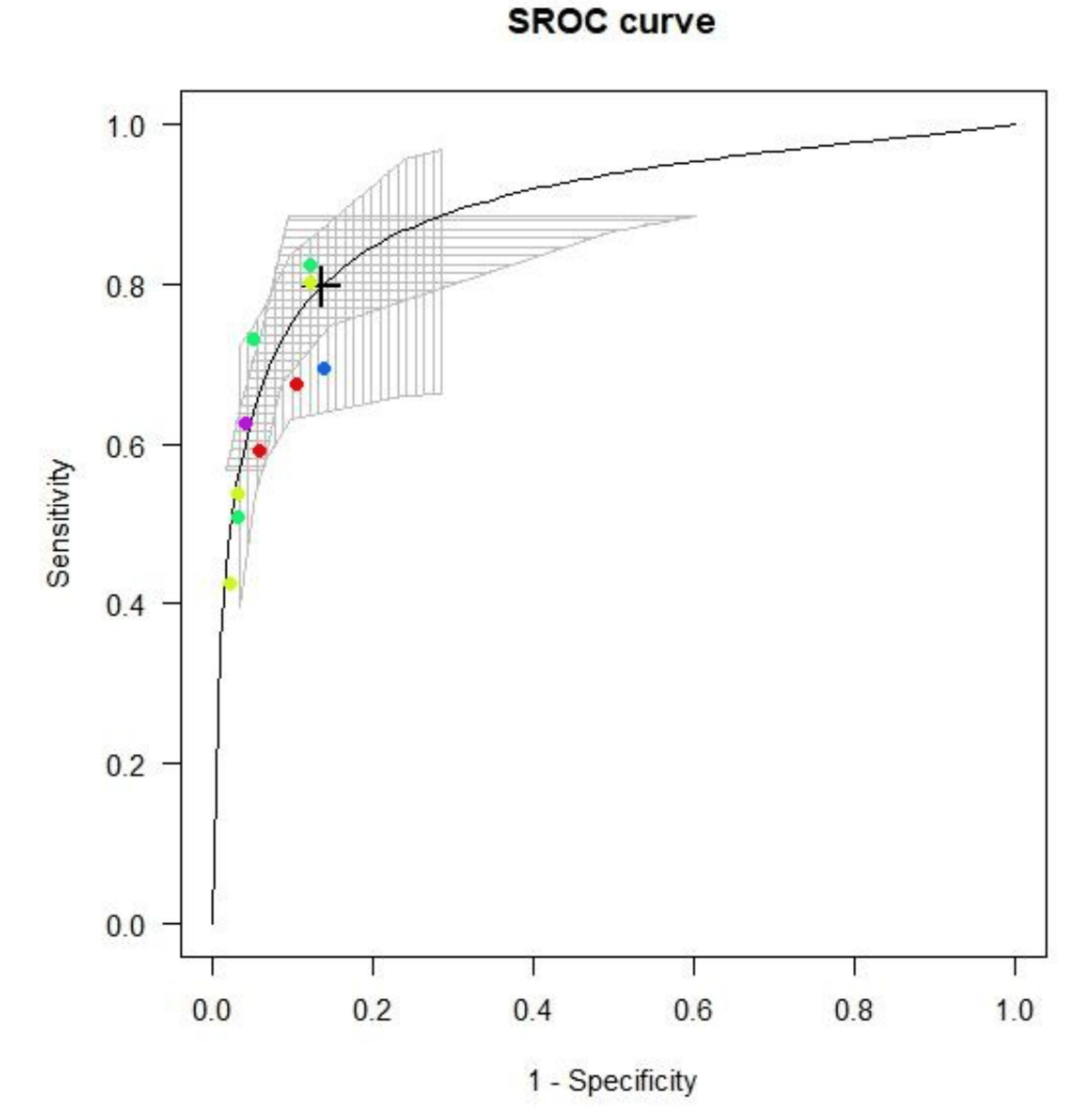
Summary ROC curve ROC: receiver operating characteristic

A summary ROC curve was generated, as shown in Figure 4. The crosshairs symbol denotes the position of the optimal threshold 1373 IU/L derived by calculation of the optimised Youden Index. Sensitivity at this threshold was 0.80 (0.65-0.89), and specificity was 0.86 (0.76-0.93). The derived optimal threshold presented here is a statistical estimate across studies and should not be interpreted as a clinically evaluated diagnostic cut-off point. 

Studies not included in the meta-analysis are as follows:

Houry et al. [35] used a threshold above the upper limit of normal and reported a sensitivity of 0.80 and a specificity of 0.67. No confidence intervals were provided. This study was excluded from the meta-analysis as it does not meet the reference standard diagnostic criteria for acute pancreatitis and thus is not similar enough in design to allow valid pooling of its results in the meta-analysis.

Husayni et al. [36] used a threshold of 306 IU/L and reported a sensitivity of 0.96 and a specificity of 0.90. No confidence intervals were provided. The mean value of urinary amylase in the case group was 1180 IU/L (standard deviation (SD): 752.9), and in the control group, 266.1 IU/L (SD: 501.9). This study was excluded from the meta-analysis because it was unclear if all patients were included in the study. Additionally, details on the control group, particularly regarding symptoms or final diagnoses, were limited. The reported outcome measures did not allow construction of a 2×2 contingency table, rendering the study unsuitable for inclusion in the pooled analysis.

Kalaiventhan et al. [37] reported a mean urinary amylase concentration of 1577.26 IU/L (SD: 457.82) in the case group and 298.13 IU/L (SD: 352.17) in the control group. The study was excluded from the meta-analysis due to multiple exclusion criteria, the use of an asymptomatic control group, and the fact that although sensitivity and specificity were mentioned, they were not actually calculated. The timing of the index test was also unclear and may have occurred after diagnosis rather than concurrently with serum sampling. Furthermore, the reported outcomes did not allow construction of a 2×2 contingency table, making the study unsuitable for inclusion in the pooled analysis.

Discussion

Urinary amylase does not demonstrate superior diagnostic accuracy to serum amylase in acute pancreatitis. As highlighted in the introduction, a purported benefit of measuring urinary amylase (beyond its role in excluding macroamylasemia) lies in its potential to assist in diagnosing delayed or atypical presentations. This is attributed to its prolonged elevation compared to serum amylase levels, which tend to normalise faster. Unfortunately, none of the studies rigorously stratified the collection of spot urine samples by time since symptom onset, so the value of urinary amylase to this end remains hypothetical. Despite the introduction of other diagnostic tests, serum amylase remains a widely utilised test in the evaluation of acute pancreatitis. For serum amylase, the normal range is typically between 28 and 100 IU/L [38], and values greater than three times the upper limit of normal are suggestive. The normal range for urinary amylase is typically around 450-460 IU/L [39], but the use of urinary amylase in the diagnosis of acute pancreatitis is not recommended in existing guidelines [6,40-42]. 

A systematic review conducted by Cochrane in 2017 [43] identified a single study from China focusing on admission quantitative urinary amylase levels among 134 patients. In this study, the diagnostic threshold was established ‘above the normal reference range’ (unspecified), yielding a sensitivity of 0.83 (95% CI: 0.65-0.94) and specificity of 0.86 (95% CI: 0.77-0.91). In comparison, the same Cochrane review reported admission serum amylase sensitivity of 0.72 (95% CI: 0.59-0.82) and specificity of 0.93 (95% CI: 0.66-0.99). Serum lipase sensitivity was 0.79 (95% CI: 0.54-0.92) with a specificity of 0.89 (95% CI: 0.46-0.99). Our findings for urinary amylase - sensitivity 0.80 (95% CI: 0.65-0.89) and specificity 0.86 (95% CI: 0.76-0.93) - are similar to those presented in the 2017 Cochrane review, although we observed lower sensitivity levels. We looked at a wide variety of study types given the paucity of diagnostic randomised trials in this field, as outlined by Cochrane in 2017. It is important to emphasise that all enzymes serve as supportive markers in the diagnosis rather than definitive indicators of acute pancreatitis [44].

Limitations and Strengths

We performed a comprehensive search of peer-reviewed literature and included all studies adhering to our review's inclusion criteria. Studies not included in the meta-analysis were reported in a narrative fashion. The study populations were drawn from a range of centres, enhancing the generalisability of our findings. Statistical heterogeneity is low; however, this may be affected by the small number of studies and amylase forming part of the reference standard. Deeks' funnel plot test indicated no significant publication bias. This would have been aided by the broad search strategy used in our review, but caution is advised as the small number of studies does limit statistical assessment of publication bias. Language bias is a risk as we only included studies published in English.

Although patients in the included studies varied in the duration of symptoms before presentation, none of the papers clearly reported the timing of sample collection in relation to symptom onset for individual cases. This precludes any conclusions being made about a role for urinary amylase in delayed or atypical presentations. In most of the studies included in this review, the index test scored a high risk of bias. This was due to amylase forming part of the diagnostic criteria for acute pancreatitis itself, which can overvalue the diagnostic performance of the enzyme. Our review consisted of a small number of cross-sectional and case-control studies, and no diagnostic randomised controlled trials assessing the utility of urinary amylase in acute pancreatitis were available. Only one such study (published in Chinese and therefore excluded by our language criteria) was included in the earlier Cochrane review. As a result, our work complements the existing review literature in this area.

## Conclusions

The diagnosis of acute pancreatitis cannot rely solely on enzyme testing, even when multiple enzymes are measured. Regardless of which enzyme is used, results must always be interpreted in conjunction with clinical evaluation and, in many cases, diagnostic imaging. Urinary amylase demonstrates good diagnostic accuracy but does not outperform serum amylase or lipase. Current evidence does not support the routine use of urinary amylase over standard serum tests in acute care. Future research should focus on the timing of urine sampling relative to symptom onset, using predefined urinary amylase thresholds, to determine whether urinary amylase has a role in diagnosing delayed or atypical presentations.
